# Supplementary material for: The fru gene specifies male cooperative behaviors in honeybee colonies
Source: Nat Commun. 2025 Dec 22;16:11203. doi: 10.1038/s41467-025-67392-2 (PMC12722765; doi:10.1038/s41467-025-67392-2)

Supplementary Information:

The *fru* gene specifies male cooperative behaviors in honeybee colonies

Sven Köhnen[1*], Pia Ulbricht[1], Alina Sturm[1], Julie Carcaud [2], Jean-Christophe Sandoz[2], Thomas Eltz[3] and Martin Beye[1*]

[1] Institute of Evolutionary Genetics, Heinrich-Heine University, Dusseldorf, Germany

[2] Evolution, Genomes, Behaviour and Ecology, Université Paris-Saclay, Gif-sur-Yvette, France

[3] Department of Animal Ecology, Evolution and Biodiversity, Ruhr University, Bochum, Germany

* Corresponding author: Sven Koehnen (svenkoehnen@outlook.de); Martin Beye (martin.beye@hhu.de)

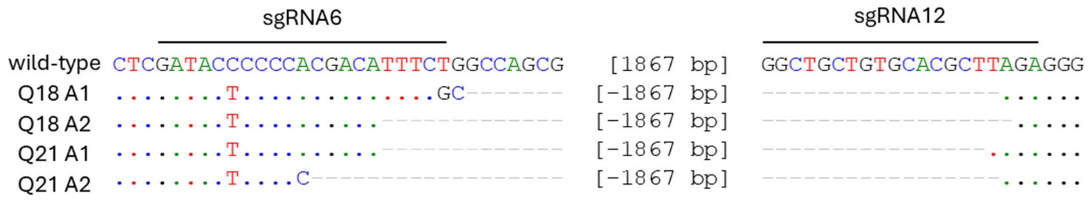

Supplementary Figure 1. Genotypes of the *fruP1*$^{-/-}$ queens. The nucleotide sequences of designated target sites of the sgRNAs are shown for queen number 18 and 21. Wildtype (wt): reference sequence.

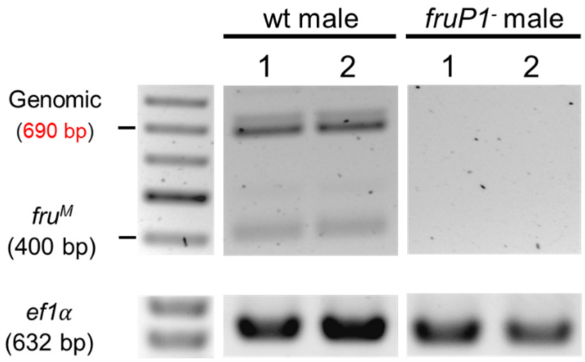

Supplementary Figure 2. Loss of P1-derived *fru*^{M} transcripts in *fruP1*$^{-}$ males. RT-PCRs of single males were size resolved and semi-quantitatively adjusted using *ef-1α* (*elongation factor 1α*) transcripts as a reference. Three technical replicates were run for each sample. Source data are provided at the end of this Supplementary Information file.

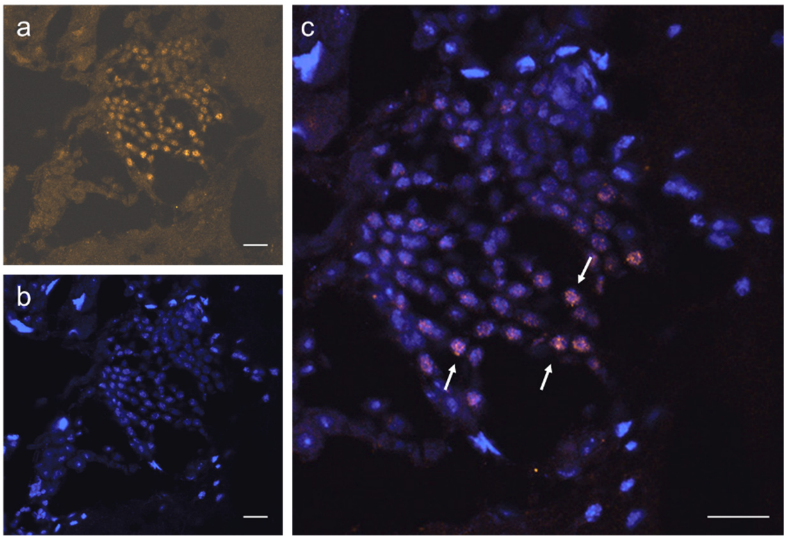

Supplementary Figure 3. Anti-Fru staining colocalizes with cell nuclei. Cryosection of a pupal brain (stage P3). Picture of a neuronal cluster in the midbrain (near the antennal lobes) that was stained with anti-Fru antibody (**a**) and Hoechst34580 (nuclei staining (**b**). **c.** Overlay of both channels. Arrows indicate examples of colocalization. Scale 20 µm.

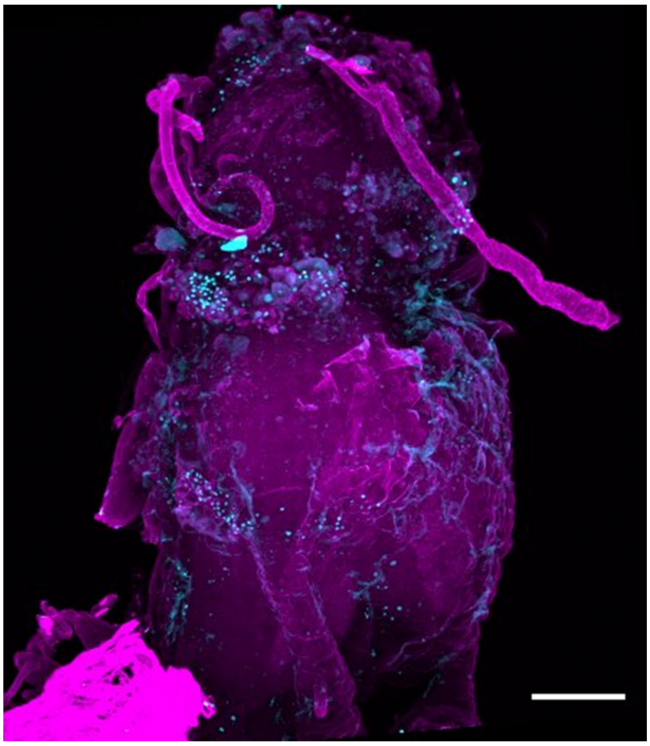

Supplementary Figure 4. Anti-Fru labeling of the male meso-metathoracic ganglion. The ganglion of an adult wt male was stained with anti-Fru (cyan) and phalloidin (magenta). n = 4 bees were examined. Scale 100 µm.

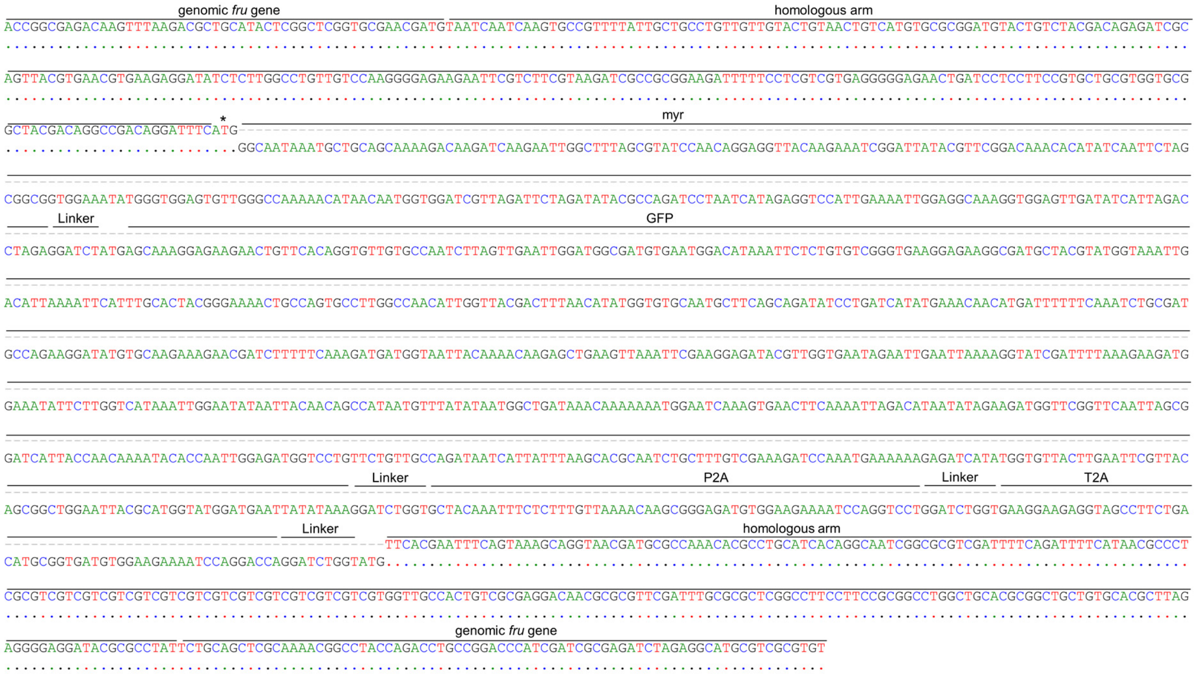

Supplementary Figure 5. The nucleotides of myrGFP-P2A-T2A coding sequence at the *fru* locus insertion site. The different coding elements were fused through linker sequences and flanked by 250 bp homologous arms to build the donor sequence that was injected into the embryos. *: translation start codon. Wildtype sequence of the *fru* locus insertion site is provided as a reference. P2A, T2A: these sequences encode the peptides mediating the separation of myrGFP and Fru^{M} proteins.

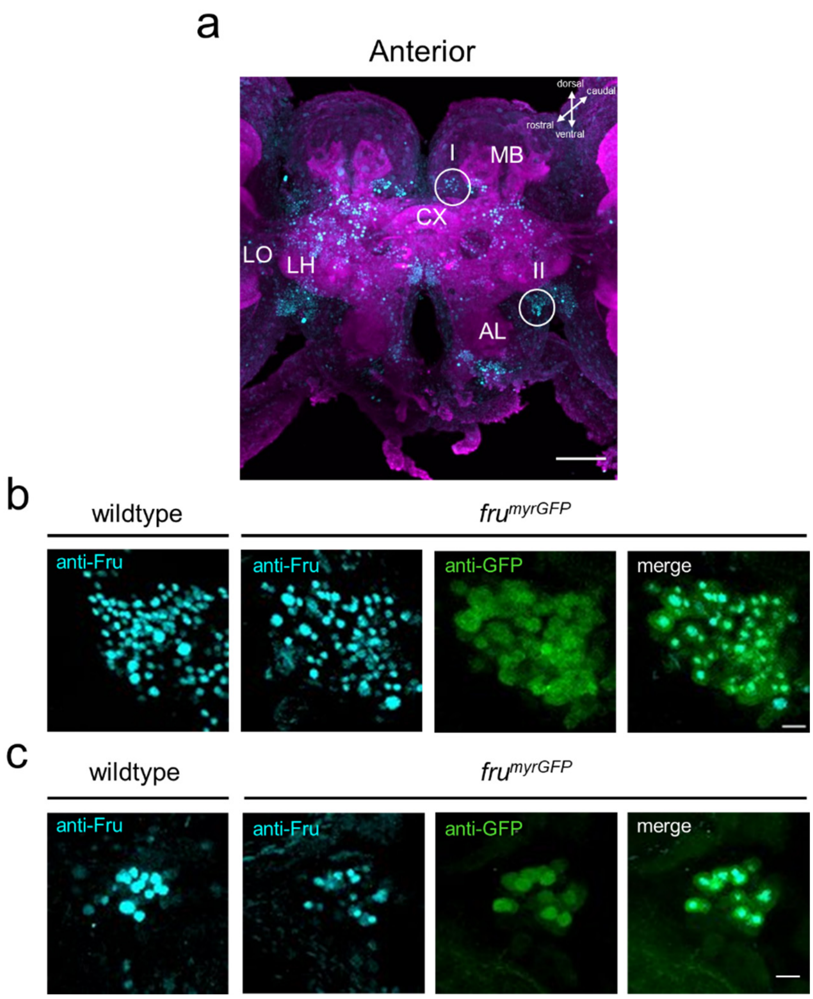

Supplementary Figure 6. Robust development and myrGFP labeling of Fru^{M} expressing cells in *fru^{myrGFP}* males. a. The position of the cluster I and II (circles) in the male midbrain that were under examination at P3 stage pupae. Cyan: anti-Fru labeling. Magenta: phalloidin (f-actin) labeling. MB: mushroom body, AL: antennal lobe, CX: central complex, LH: lateral horn, LO: lobula. Scale 100 µm. **b.** Anti-Fru (cyan) and anti-myrGFP (green) labeling of neurons in cluster I of *fru^{myrGFP}* male. Wildtype: control wt males. Scale 10 µm. **c.** Anti-Fru (cyan) and myrGFP (green) labeling of neurons in cluster II of *fru^{myrGFP}* males. Wildtype: control wt males. n = 6 bees were examined for each cluster. Scale 10 µm.

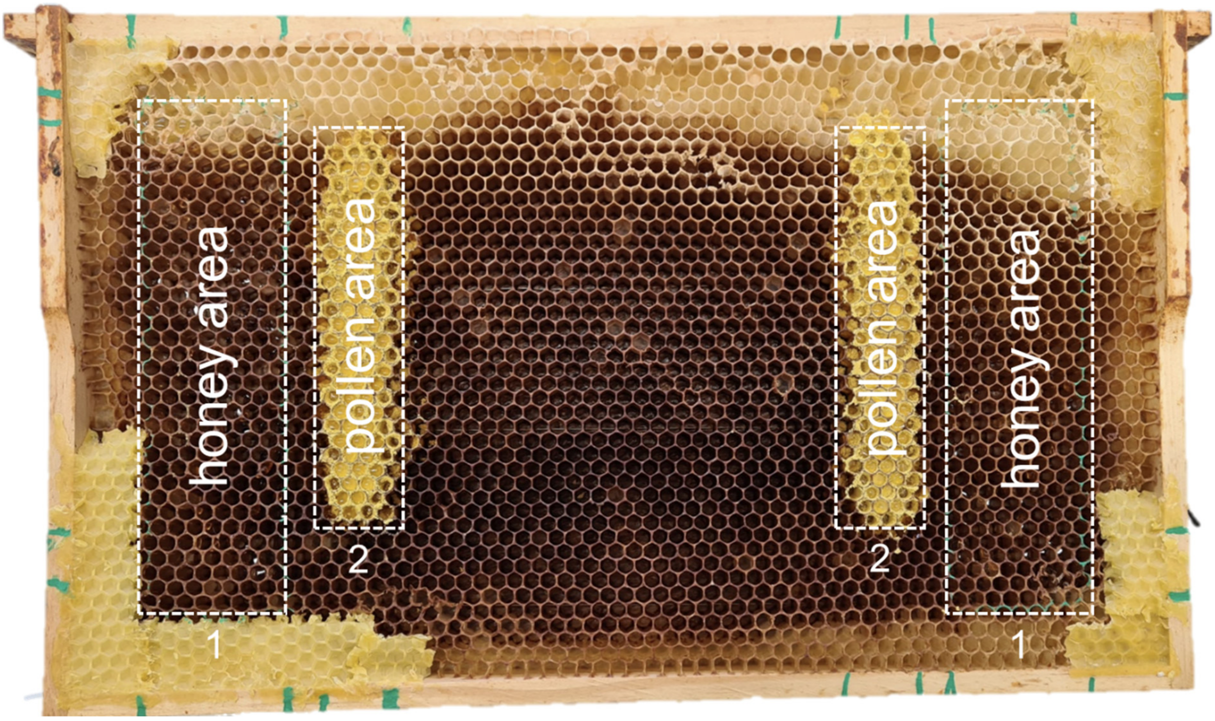

Supplementary Figure 7. **Standard comb for the behavioral tracking of male behaviors in the colony.** The same amount of sugar solution ("honey area": 225 cells each filled with 200 µl of 70% saccharose solution) and pollen ("pollen area": the cells were filled with 15 g of ground pollen) were provided at the same location in the different biological replicates.

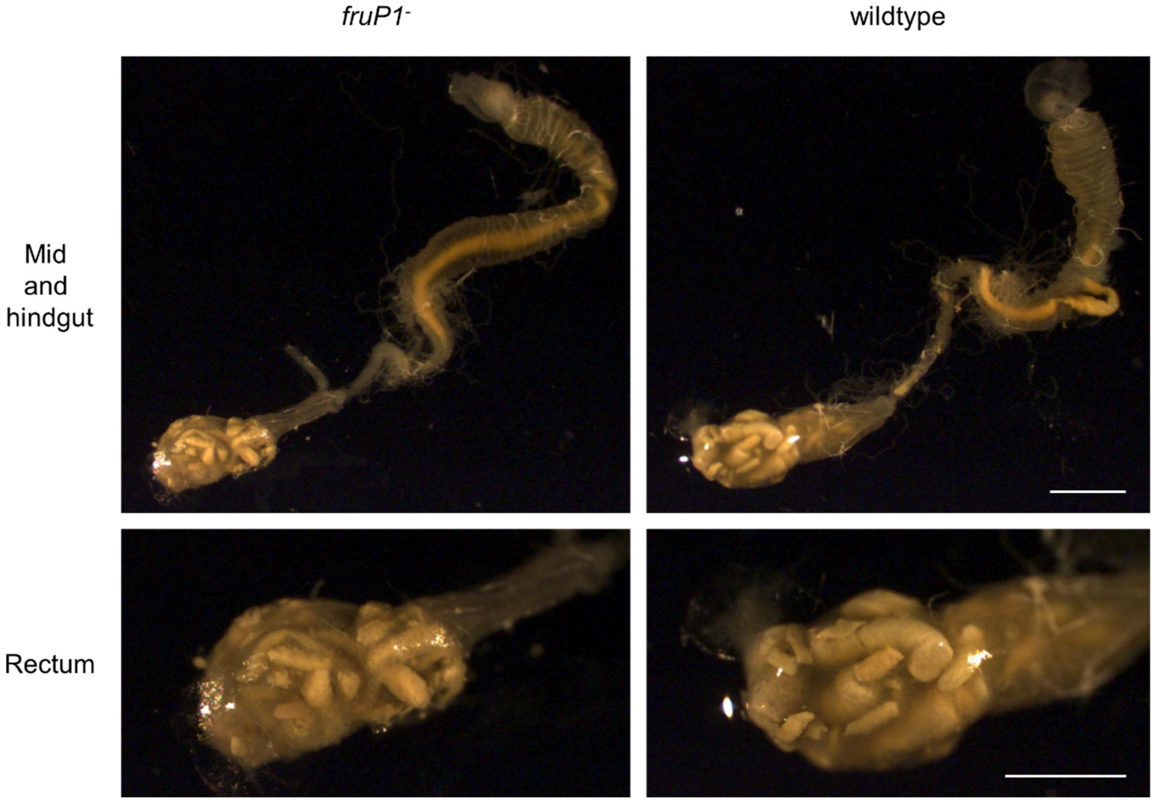

Supplementary Figure 8. Pollen grains were absent in the gut of *fruP1*$^{-}$ and wt males. Dissected guts of 2 days old *fruP1*$^{-}$ (n = 8) and wt (n = 7) males that were kept on comb with honey and pollen for 24 hours. No pollen grains were detected in these guts. Scale bar: 2 mm.

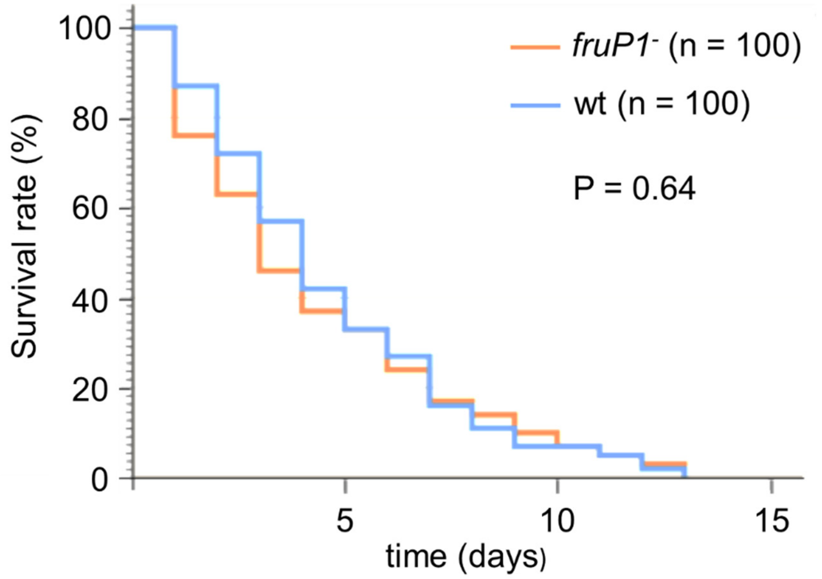

Supplementary Figure 9. Survival of *fruP1*⁻ and wt males held in the laboratory. P value of log-rank test is indicated.

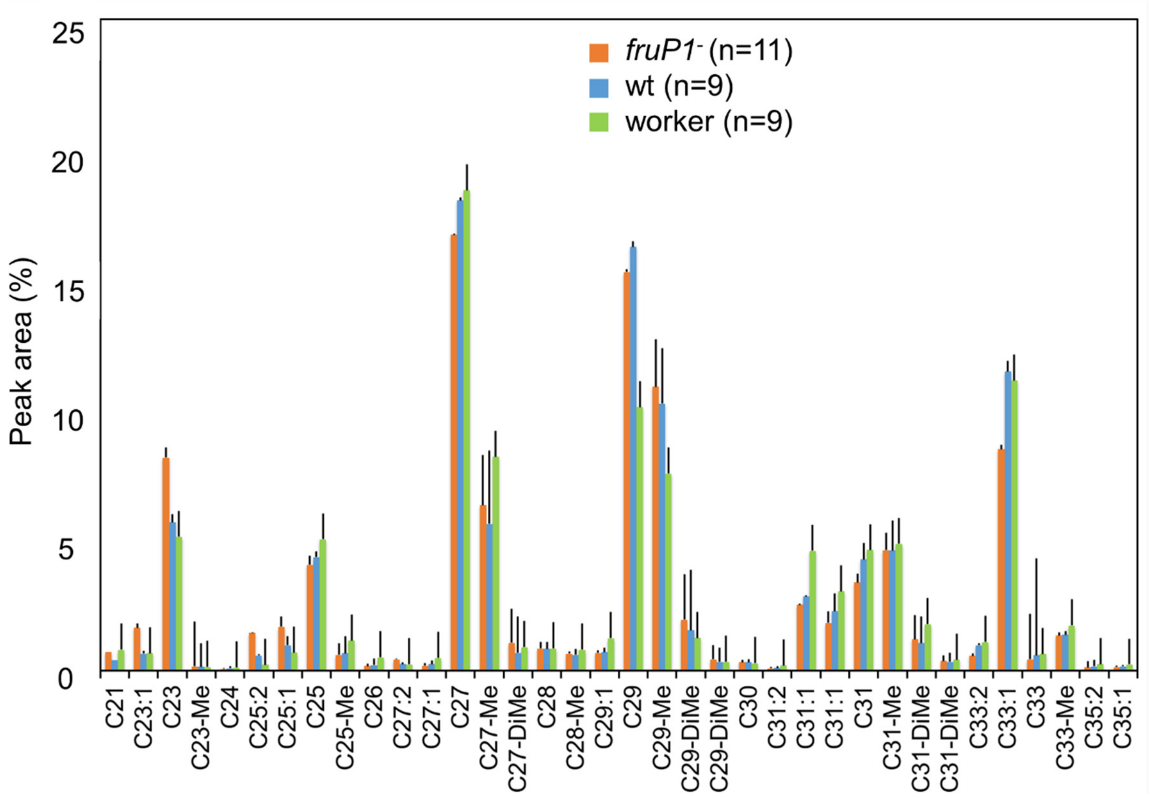

Supplementary Figure 10. Relative abundance (integrated ion currents) of cuticular hybrocarbons (CHC) as listed by retention time on a DB-5ms capillary column. The overall profiles were significantly different between *fruP1⁻* and wt males due to small accumulating differences (ANOSIM: R = 0.44, P < 0.002). This difference was not associated with specific CHC classes (alkanes, alkenes, branched compounds). The abbreviations for cuticular hydrocarbons on the x-axis contain the total number of carbon atoms in the molecule (Cx), the presence of one or two double bonds in unsaturated alkenes (:1) or alkadienes (:2), and the presence of one (-Me) or two (-DiMe) methyl groups in branched hydrocarbons. n = 11 *fruP1⁻* males, n = 9 wt males and n = 9 worker bees were examined.

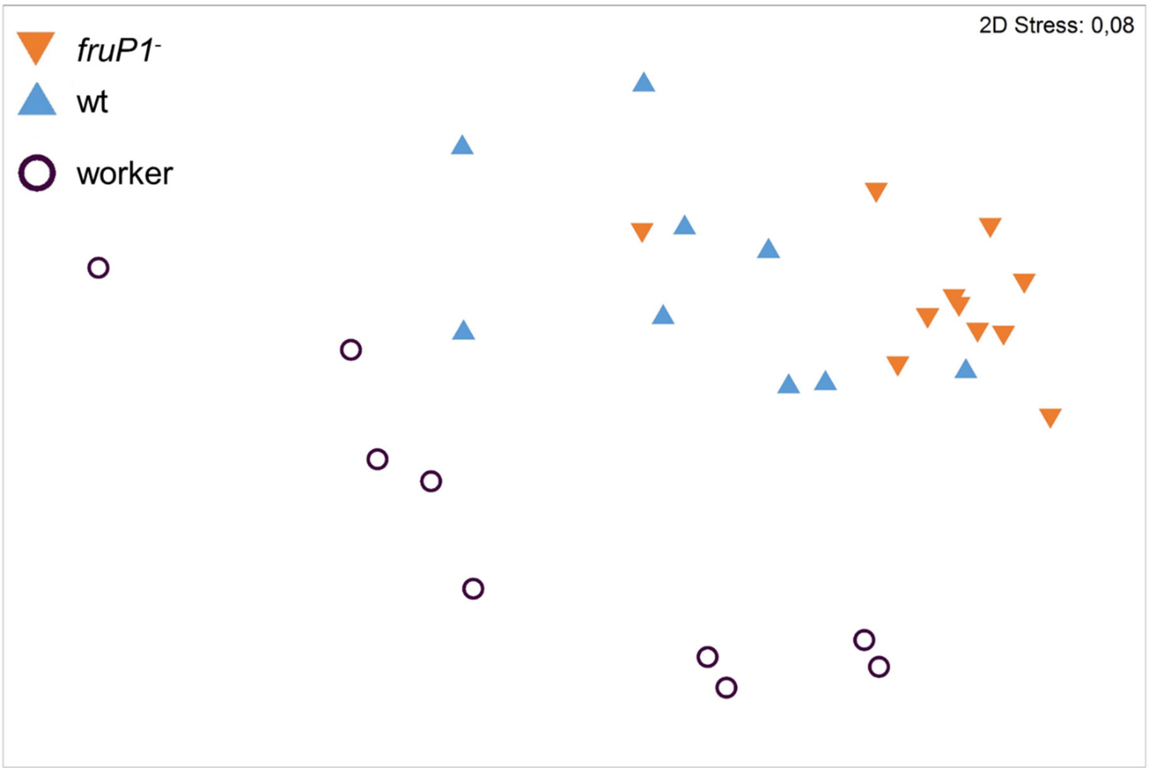

Supplementary Figure 11. The individual profiles show overlap between *fruP1-* and wt males. Cuticular hydrocarbon (CHC) profiles are presented as a two-dimensional Multidimensional Scaling (MDS) plot based on standardized, square-root transformed Bray-Curtis distances. The stress of 0.08 indicates a good representation of the underlying dissimilarity matrix.

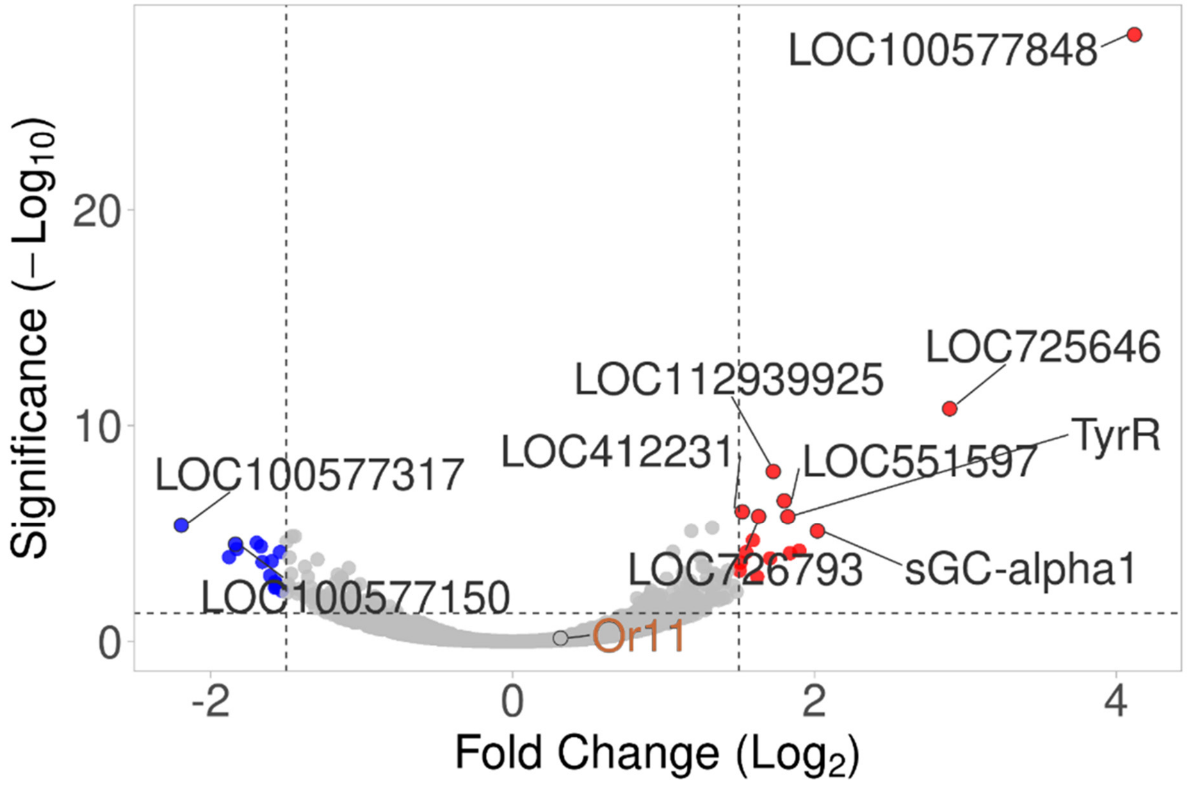

Supplementary Figure 12. Transcriptome differences in the antennae of *fruP1-* and wt males. Genes with negative log2 fold-change values (left) were more highly expressed in wt males, and genes with positive log2 fold-change values (right) were more highly expressed in *fruP1*$^{-}$ males. Red and blue circles indicate genes with significantly different expressions (adjusted P-value, Padj < 0.05, Wald test, DESeq2 and a log2 (fold change) value ≥ 1.5). n = 3 *fruP1*$^{-}$ male biological replicates and n = 3 wt male biological replicates were performed. Each such replicate were pools that derived from 5 individuals. There was no chemosensory receptor gene differently expressed between *fruP1*$^{-}$ and wt males, which includes the highly male-specific expressed OR11 gene (orange). The 10 most differentially expressed genes are assigned.

Supplementary Table 1: List of sex-specific spliced genes in the pupal brain and the subset of genes that encode a transcription factor

Gene ID or name	Transcription factor annotation
LOC100576463	no
LOC727131	no
Cht5	no
LOC551437	no
LOC100576773	no
SP5	no
LOC413942	no
LOC408414	no
LOC727007	no
LOC100578735	no
SP5	no
LOC412885	no
LOC551170	no
fru	yes
GMCOX1	no
LOC724916	no
LOC724172	no
Fem	no
LOC551170	no
cul-2	no
glu	yes
LOC410261	no
LOC413942	no
LOC100578685	no
dsx	yes

Supplementary Table 2: The frequencies of male-specific *fruP1* spliced transcripts in *fem* RNAi females

	Number of individuals		
	fem siRNA females	wt females	wt males
Am-fru^{M}	3	0	5
Am-fru^{F}	0	5	0
Am-fru^{M} + *Am-fru*^{F}	12	0	0

Supplementary Table 3: The proportion of anti-Fru labeled cells expressing the GFP protein.

Cluster I

Individual	Number of cells expressing Fru^M	Number of cells expressing GFP	Percentage of cells expressing Fru^M and GFP
A1	21	18	86 %
A2	24	24	100 %
A3	25	25	100 %
A4	26	24	92 %
A5	22	22	100 %
A6	21	21	100 %

Cluster II

Individual	Number of cells expressing Fru^M	Number of cells expressing GFP	Percentage of cells expressing Fru^M and GFP
A1	69	69	100 %
A2	76	76	100 %
A3	81	81	100 %
A4	73	73	100 %
A5	89	89	100 %
A6	84	84	100 %

Supplementary Table 4: The number of bouts of cell entering activities in an hour.

	Number of bouts in	
Genotype	Pollen Area	Other areas
fruP1^{-}	30	5
wt	1	2

Supplementary Table 5: Proboscis extension response (PER) to honey

	Numbers of bees	# responding	# non responding	
fruP1-	20	14 (70%)	6 (30%)	P = 0.51
wildtype control	20	11 (55%)	9 (45%)	

(Fisher exact test, df = 1)

Source data of Supplementary Figure 1:

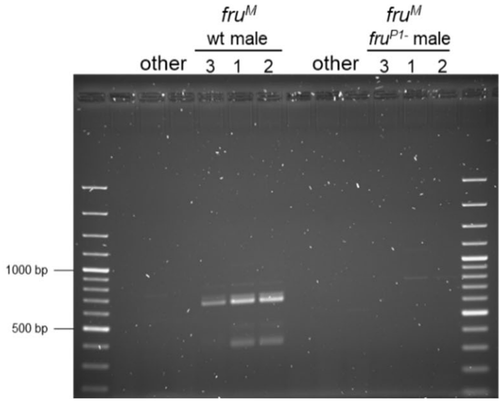

Sup. Fig. 2

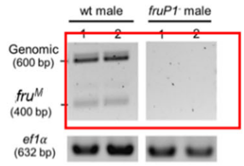

ef1α

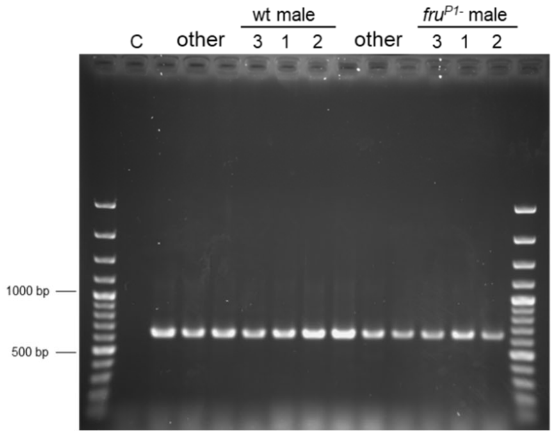

Sup. Fig. 2

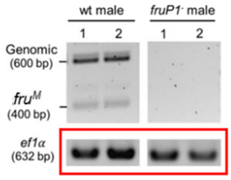

Supplement: Supplementary file 1 — Supplementary Information [file 41467_2025_67392_MOESM1_ESM.pdf]
