# Supplementary material for: The fru gene specifies male cooperative behaviors in honeybee colonies
Source: Nat Commun. 2025 Dec 22;16:11203. doi: 10.1038/s41467-025-67392-2 (PMC12722765; doi:10.1038/s41467-025-67392-2)
Supplement: Supplementary file 2 — Description of Additional Supplementary Files [file 41467_2025_67392_MOESM2_ESM.docx]

**Description of Additional Supplementary Files**

*(In bold letters the title shown)*

**File name: Supplementary Movie 1**

**Phalloidin and anti-Fru stained male; midbrain**

Description: A middle brain z-stack of a wildtype male that is stained with phalloidin (magenta) and anti-Fru (cyan).

**File name: Supplementary Movie 2**

**Phalloidin and anti-Fru stained worker female bee, midbrain**

Description: A middle brain z-stack of a wildtype worker bee that is stained with phalloidin (magenta) and anti-Fru (cyan).

**File name: Supplementary Movie 3**

**Phalloidin and anti-Fru stained *fruP1*^-^ male, midbrain**

Description: A middle brain z-stack of a *fruP1^-^* male that is stained with phalloidin (magenta) and anti-Fru (cyan).

**File name: Supplementary Movie 4**

**Phalloidin and anti-GFP stained *fruP1^myrGFP^* male, midbrain (anterior to medial region)**

Description: A middle brain z-stack of the anterior to medial region of a *fruP1^myrGFP^* male that is stained with phalloidin (magenta) and anti-GFP (green).

**File name: Supplementary Movie 5**

**Phalloidin and anti-GFP stained *fruP1^myrGFP^* male, midbrain (posterior region)**

Description: A middle brain z-stack of the posterior region of a *fruP1^myrGFP^* male that is stained with phalloidin (magenta) and anti-GFP (green).

**File name: Supplementary Movie 6**

**anti-GFP stained *fruP1^myrGFP^* male, midbrain (anterior to medial region)**

Description: A middle brain z-stack of the anterior to medial region of a *fruP1^myrGFP^* male that is stained with phalloidin and anti-GFP (green). Only the anti-GFP staining is shown. Arrows and arrowheads mark structures shown in Fig. 4.

**File name: Supplementary Movie 7**

**anti-GFP stained *fruP1^myrGFP^* male, midbrain (posterior region)**

Description: A middle brain z-stack of the posterior region of a *fruP1^myrGFP^* male that is stained with phalloidin and anti-GFP (green). Only the anti-GFP staining is shown. Arrows and arrowheads mark structures shown in Fig. 4.

**File name: Supplementary Movie 8**

**anti-GFP stained *fruP1^myrGFP^* male, optical lobe (anterior to medial region)**

Description: Optical lobe z-stack of the anterior to medial region of a *fruP1^myrGFP^* male that is stained with phalloidin (magenta) and anti-GFP (green). Only the anti-GFP staining is shown.

**File name: Supplementary Movie 9**

**anti-GFP stained *fruP1^myrGFP^* male, optical lobe (posterior region)**

Description: Optical lobe z-stack of the posterior region of a *fruP1^myrGFP^* male that is stained with phalloidin (magenta) and anti-GFP (green). Only the anti-GFP staining is shown.

**File name: Supplementary Movie 10**

**Phalloidin and anti-GFP stained *fruP1^myrGFP^* worker bee, midbrain**

Description: A middle brain z-stack of a *fruP1^myrGFP^* worker bee that is stained with phalloidin (magenta) and anti-GFP (green).

**File name: Supplementary Movie 11**

**anti-GFP stained *fruP1^myrGFP^* worker bee, midbrain**

Description: A middle brain z-stack of a *fruP1^myrGFP^* worker bee that is stained with phalloidin (magenta) and anti-GFP (green). Only the anti-GFP staining is shown. Arrows and arrowheads mark structures shown in Fig. 4.

**File name: Supplementary Movie 12**

**Phalloidin and anti-GFP stained** **wt male bee, midbrain**

Description: A middle brain z-stack of a wt male bee that is stained with phalloidin (magenta) and anti-GFP (green).

**File name: Supplementary Movie 13**

**anti-GFP stained wt male bee, midbrain**

Description: A middle brain z-stack of a wt male bee that is stained with phalloidin (magenta) and anti-GFP (green). Only the anti-GFP staining is shown. Arrows and arrowheads mark structures shown in Fig. 4.

**File name: Supplementary Movie 14**

**Example of a *fruP1^-^* male bee displaying begging behavior**

Description: Example of a *fruP1^-^* male bee (highlighted with the ID # 18) showing food begging behavior. The male bee approaches the head of a worker bee with its antennae while extending the proboscis.

**File name: Supplementary Movie 15**

**Example of a *fruP1^-^* male bee displaying trophallaxis behavior**

Description: Example of a *fruP1^-^* male bee (highlighted with the ID # 44) showing trophallaxis behavior. The male bee is being fed by the worker bee. Glossa of the worker is protruded between mandibels. Sporadically, the male bee contacts the worker bee with its forelegs during this behavior.

**File name: Supplementary Movie 16**

**Example of a wt male bee displaying begging behavior**

Description: Example of a wt male bee (highlighted with the ID # 47) showing food begging behavior. The male bee approaches the head of a worker bee with its antennae while extending the proboscis.

**File name: Supplementary Movie 17**

**Example of a wt male bee displaying trophallaxis behavior**

Description: Example of a wt male bee (highlighted with the ID # 16) showing trophallaxis behavior. The male bee is being fed by the worker bee. Glossa of the worker is protruded between mandibels. Sporadically, the male bee contacts the worker bee with its forelegs during this behavior.

**File name: Supplementary Movie 18**

**Example of a *fruP1^-^* male bee displaying an approaching behavior towards worker bee**

Description: Example of a *fruP1^-^* male bee (highlighted with the ID # 27) that contacts a worker bee. The male bee approaches the abdomen of a worker bee with its antennae while extending the proboscis.

**File name: Supplementary Movie 19**

**Example of a wt male bee displaying an approaching behavior towards a worker bee**

Description: Example of a wt male bee (highlighted with the ID # 50) that contacts a worker. The male bee approaches the abdomen of a worker bee with its antennae while extending the proboscis.

**File name: Supplementary Movie 20**

**Example of a *fruP1^-^* male bee displaying an approaching behaviour towards male**

Description: Example of a *fruP1^-^* male bee (highlighted with the ID # 48) that contacts another male. The male bee approaches the head of another male with its antennae while extending the proboscis.

**File name: Supplementary Movie 21**

**Example of a wt male bee trophallaxis behaviour towards male**

Description: Example of a wt male bee (highlighted with the ID # 18) that contacts another male. The male bee approaches the head of another male with its antennae while extending the proboscis.

**File name: Supplementary Movie 22**

**Example of a *fruP1^-^* male bee performing cleaning behavior**

Description: Example of a *fruP1^-^* male bee (highlighted with the ID # 32) that performs self-cleaning behavior. The male bee brushes parts of its head with its forelegs.

**File name: Supplementary Movie 23**

**Example of a wt male bee performing cleaning behavior**

Description: Example of a wt male bee (highlighted with the ID # 44) that performance self-cleaning behavior. The male bee brushes parts of its head with its forelegs.

**File name: Supplementary Movie 24**

**Example of a *fruP1^-^* male bee displaying resting behavior**

Description: Example of a *fruP1^-^* male bee (highlighted with the ID # 25) that displays resting behavior. The male bee rests. Antennae are lowered and wings are folded.

**File name: Supplementary Movie 25**

**Example of a wt male bee displaying resting behavior**

Description: Example of a wt male bee (highlighted with the ID # 22) that displays resting behavior. The male bee rests. Antennae are lowered and wings are folded.

**File name: Supplementary Movie 26**

**Example of a *fruP1^-^* male bee that displays cell entering activity**

Description: Example of a *fruP1^-^* male bee (highlighted with the ID # 8), that displays cell entering behavior. The male’s head is in the cell and the antennae are not visible.

**File name: Supplementary Movie 27**

**Example of a wt male bee that displays cell entering activity**

Description: Example of a wt male bee (highlighted with the ID # 33), that displays cell entering behavior. The male’s head is in the cell and the antennae are not visible.

**File name: Supplementary Movie 28**

**Example of a *fruP1^-^* male bee that displays bouts of cell entering activities**

Description: Example of a *fruP1^-^* male bee (highlighted with the ID # 26), that displays bouts cell entering behavior in the pollen area. The male bee repeatedly enters the cells in short time intervals.

**File name: Supplementary Movie 29**

**Example of a wt male bee that displays bouts of cell entering activities**

Description: Example of a wt male bee (highlighted with the ID # 2) that displays bouts cell entering behavior in the area harbouring empty cells. The male bee repeatedly enters the empty cells in short time intervals.

**File name: Supplementary Movie 30**

**Optical sections of a *fruP1^-^* male bee midbrain stained with phalloidin.**

Description: A middle brain z-stack of a *fruP1^-^* male that is stained with phalloidin and anti-Fru. Only the phalloidin staining (magenta) is shown.

**File name: Supplementary Movie 31**

**Optical sections of a wt male bee midbrain stained with phalloidin.**

Description: A middle brain z-stack of a wt male that is stained with phalloidin and anti-Fru. Only the phalloidin staining (magenta) is shown.
