# Supplementary figures and images for: The fru gene specifies male cooperative behaviors in honeybee colonies
Source: Nat Commun. 2025 Dec 22;16:11203. doi: 10.1038/s41467-025-67392-2 (PMC12722765; doi:10.1038/s41467-025-67392-2)

Fig. 1 e

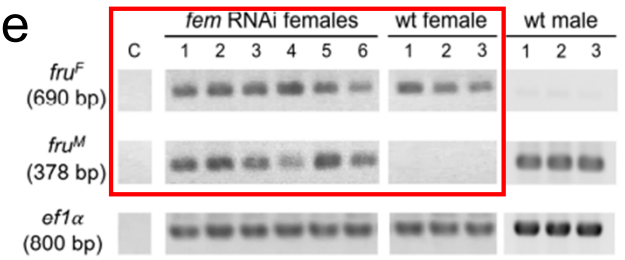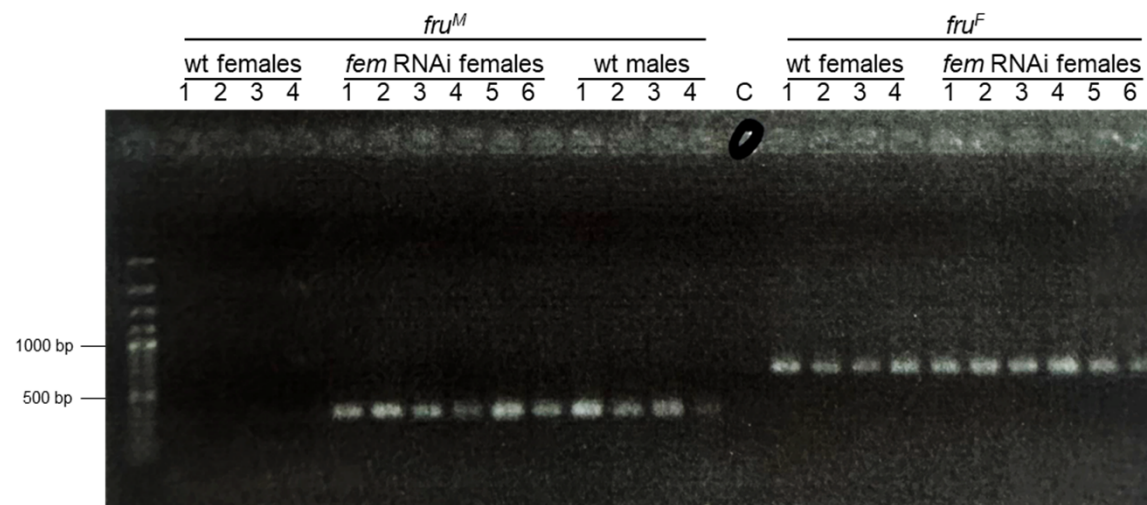

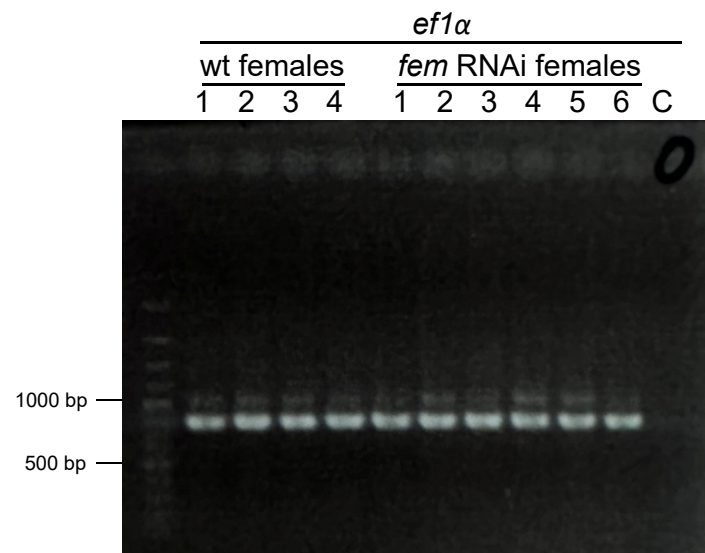

Fig. 1 e

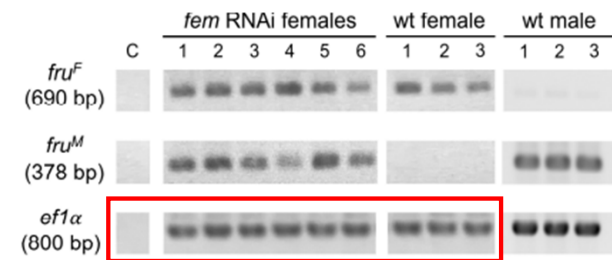

Fig. 1 e

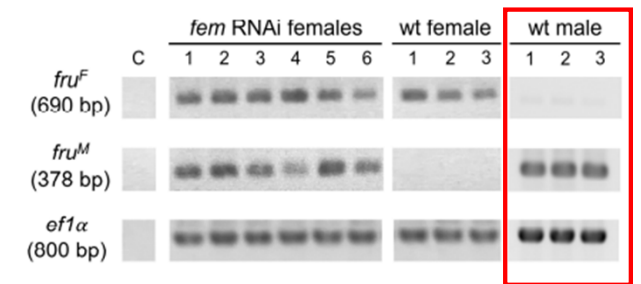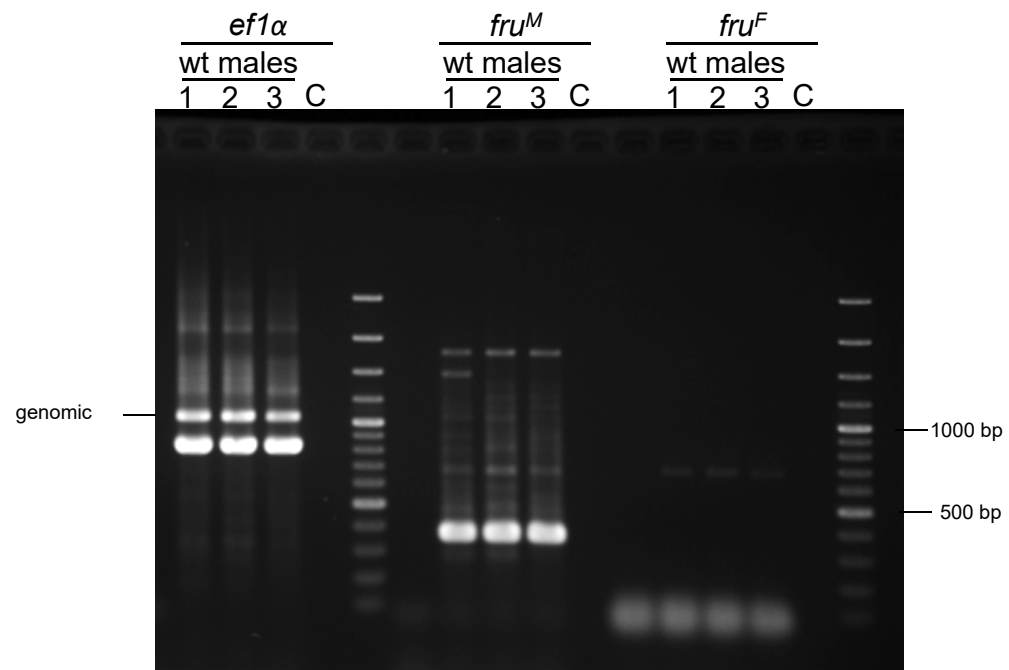

Fig. 1 f

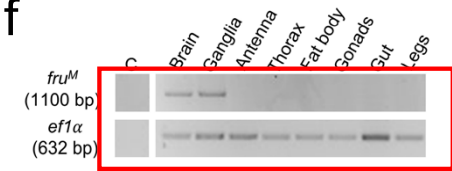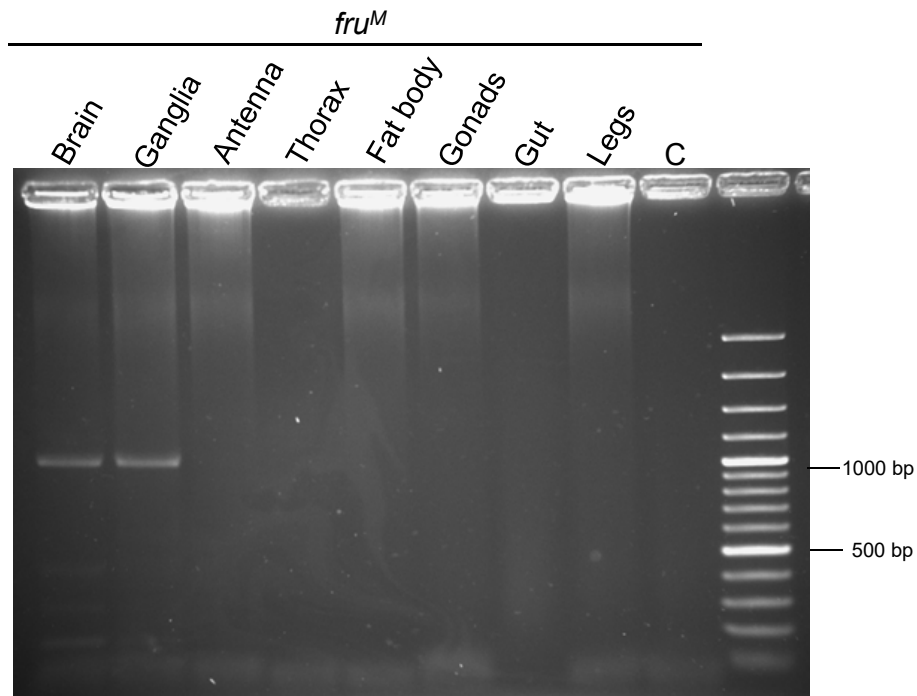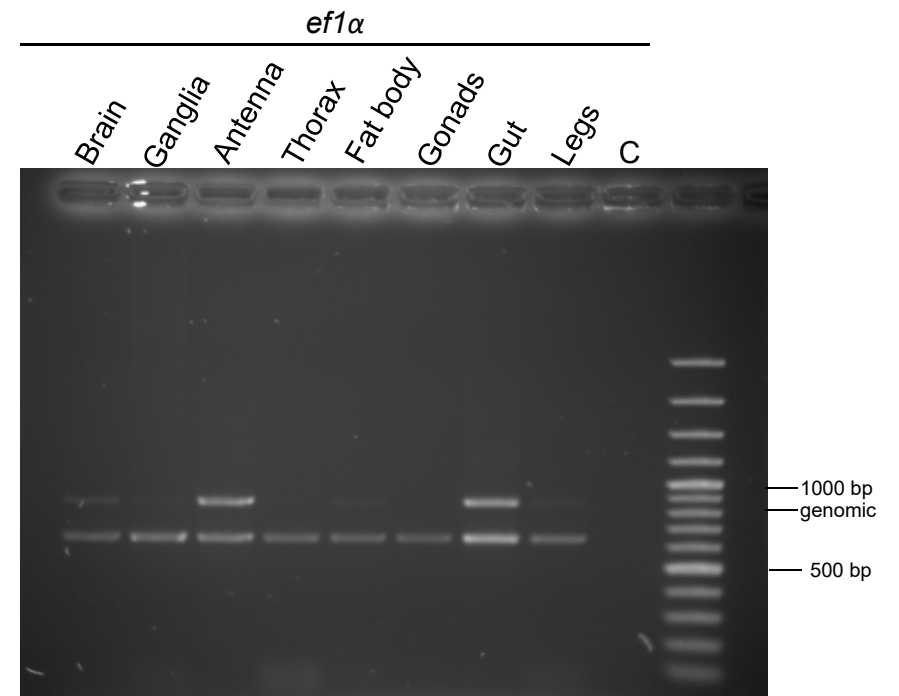

Fig. 1 g

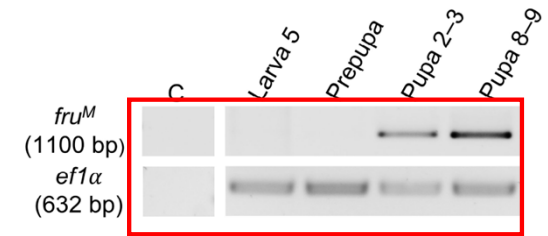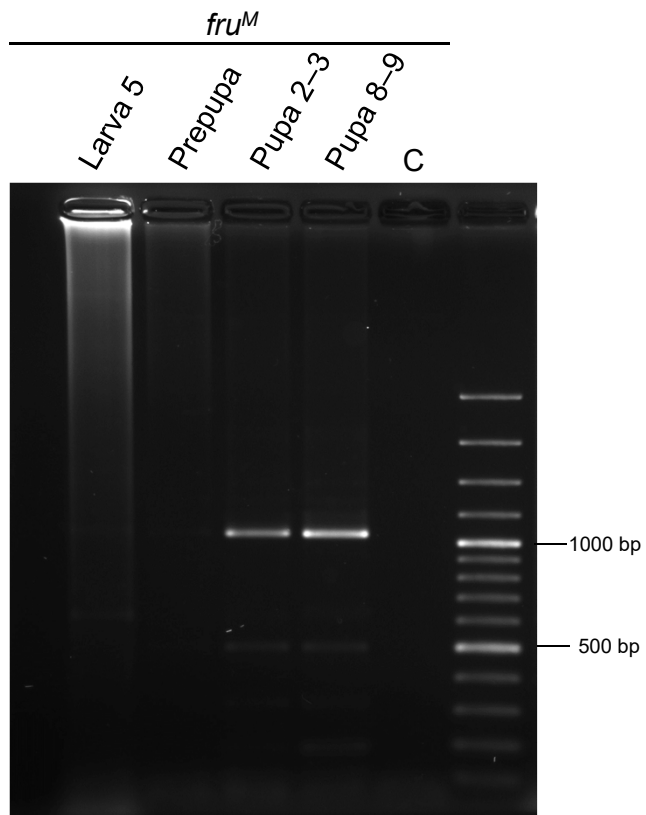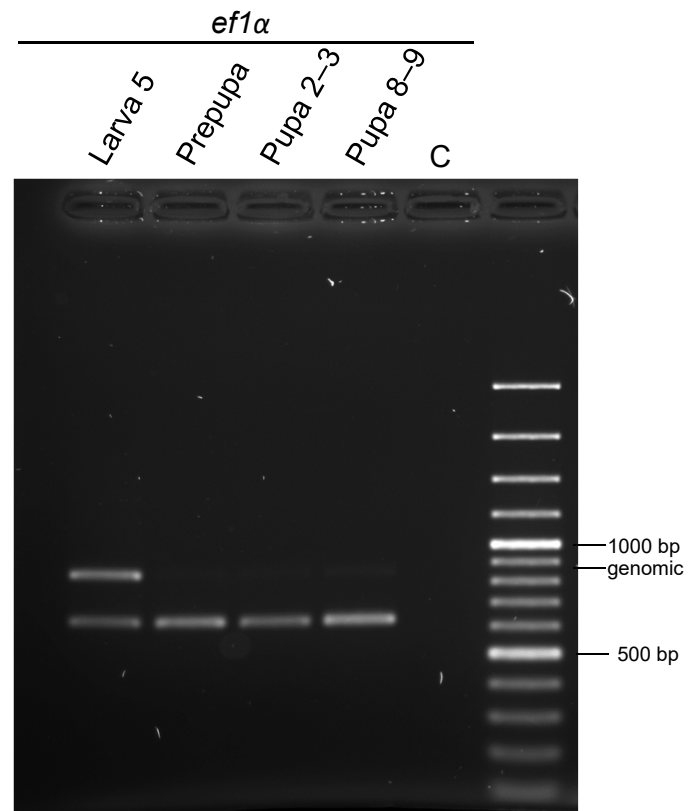

Supplement: Supplementary file 6 — Source data [file 41467_2025_67392_MOESM6_ESM.zip › Source Data file Gel.pdf]
